# Association Between Wearable Device Adoption and Health-Related Lifestyle Behaviors: Retrospective Cohort Study

**DOI:** 10.2196/88276

**Published:** 2026-05-25

**Authors:** Eunjeong Choi, Seoyeong Choi, Suk-Yong Jang

**Affiliations:** 1 Department of Public Health, Graduate School, Yonsei University, Seoul, Republic of Korea; 2 Department of Biohealth Industry, Policy Analysis Division, Graduate School of Transdisciplinary Health Science, Yonsei University, 50 Yonsei-ro, Seodaemun-gu, Seoul, 03722, Republic of Korea, 82 2 2228 1511, 82 2 392 7734; 3 Department of Health Policy and Management, Graduate School of Public Health, Yonsei University, Seoul, Republic of Korea

**Keywords:** wearable electronic devices, digital health, self-monitoring, health behavior, physical activity, healthy aging

## Abstract

**Background:**

Wearable devices are increasingly adopted for personal health monitoring, but evidence on their long-term associations with health-related lifestyle behaviors in real-world population settings remains limited.

**Objective:**

This study examined the longitudinal association between wearable device adoption and engagement in health-related lifestyle behaviors using a nationally representative panel dataset from South Korea.

**Methods:**

We analyzed lifestyle activity outcomes from the 2016 and 2022 waves and wearable device ownership from the 2017 and 2022 waves of the Korea Media Panel survey. Health-related lifestyle behaviors in the physical, social, and cultural domains were operationalized as estimated annual activity counts based on self-reported frequency measures. We used a difference-in-differences framework with generalized estimating equations to compare changes in these behaviors between new wearable adopters and nonadopters adjusting for demographic and socioeconomic characteristics. Relative changes were estimated using Poisson models with a log link, and subgroup analyses were conducted to explore variation across sociodemographic groups. As a sensitivity analysis, inverse probability of treatment weighting was additionally applied to assess the robustness of the findings to observed baseline imbalance.

**Results:**

Wearable device adoption was associated with greater increases in total, physical, and cultural health-related lifestyle activities over time. In the difference-in-differences model, adopters showed greater relative increases in total activity (rate ratio [RR] 1.24, 95% CI 1.08-1.35), physical activity (RR 1.36, 95% CI 1.12-1.64), and cultural activity (RR 1.78, 95% CI 1.31-2.42) than nonadopters. Subgroup analyses showed limited evidence of consistent heterogeneity and should be interpreted cautiously. Sensitivity analyses using inverse probability of treatment weighting showed overall patterns broadly similar to those of the primary analyses.

**Conclusions:**

In this nationally representative panel study, wearable device adoption was associated with greater increases in total, physical, and cultural health-related lifestyle activities over time, whereas no clear association was observed for social activity. These findings should be interpreted as associative rather than causal given the observational design and the inability to directly assess parallel trends.

## Introduction

The World Health Organization defines health not merely as the absence of disease but as a state of physical, mental, and social well-being [1]. This broader perspective suggests that health-related behaviors should be understood not only as isolated actions but also as part of wider lifestyle patterns shaped by social and structural contexts. Although much health behavior research has focused on single behaviors or narrow clusters of behaviors, health lifestyle theory emphasizes that behaviors are socially patterned and tend to cluster in ways that reflect broader orientations toward health and daily life [2-4]. Within this context, wearable devices have gained increasing attention as tools for personal health monitoring and self-tracking. Prior studies suggest favorable associations between wearable devices and physical activity, and in some contexts, wearable-related interventions may also be linked to motivation, reassurance, or perceived support [5-7]. However, much of the existing evidence comes from randomized trials or short-term studies conducted under relatively controlled conditions [8,9]. As a result, less is known about how wearable device adoption is associated with broader health-related lifestyle behaviors over longer periods in real-world population settings.

Self-determination theory may provide one possible framework for interpreting how wearable devices could be related to behavior [10]. For example, functions such as goal setting, feedback, and self-monitoring may support feelings of autonomy or competence for some users, and social features may foster relatedness in certain contexts. However, these mechanisms were not directly measured in this study. Accordingly, we draw on this framework as a conceptual reference rather than as a tested explanatory model. South Korea provides a relevant setting for examining wearable device adoption and health-related lifestyle behaviors because it has a rapidly increasing proportion of older adults, high digital connectivity, and growing public health interest in promoting healthy aging and everyday health management [11,12]. These features make South Korea an informative context for studying whether wearable device adoption is associated with changes in lifestyle-related activities in a real-world setting.

Using nationally representative panel data from South Korea, this study examined the longitudinal association between wearable device adoption and health-related lifestyle behaviors, operationalized as physical, social, and cultural activities. By following individuals over time, we aimed to assess whether changes in these activities differed between new wearable adopters and nonadopters.

## Methods

### Data Source

This study used data from the 2016, 2017, and 2022 waves of the Korea Media Panel survey. Lifestyle activity outcomes were measured in 2016 and 2022, whereas wearable device ownership was assessed in 2017 and 2022. The Korea Media Panel is a nationally representative longitudinal survey administered by the Korea Information Society Development Institute and funded by the Korean government [13]. The panel follows the same households and individuals over time to assess changes in media environments and media-related behaviors. Data are collected annually through face-to-face interviews from more than 5000 households and individuals across 17 regions in South Korea. The survey includes information on media device ownership, communication service subscriptions, expenditures, and selected psychosocial and lifestyle-related measures. This study followed the STROBE (Strengthening the Reporting of Observational Studies in Epidemiology) guidelines (Checklist 1).

### Study Population

We used the 2016 and 2022 waves as the baseline and follow-up measurements, respectively, for the lifestyle activity outcomes, whereas wearable device ownership in 2017 was used to establish wearable nonownership before potential adoption. Among 8847 participants surveyed at baseline, we excluded those who reported wearable device ownership in 2017 (n=132, 1.5%); those younger than 20 years (n=1278, 14.4%); and those who did not participate in the 2022 survey or had missing data on the exposure, outcomes, or covariates used in the analysis (n=3182, 36.0%). The final analytic sample included 5507 participants with complete data at both time points. Because exclusion due to nonparticipation or missing data was substantial, we describe this as a complete-case analysis.

### Exposure Variable

The exposure of interest was new wearable device adoption between 2017 and 2022. Participants were classified as new adopters if they did not own a wearable device in 2017 but reported wearable device ownership in 2022. Participants who did not own a wearable device in either 2017 or 2022 were classified as nonadopters.

### Outcome Variables

Health-related lifestyle behaviors were assessed using self-reported frequency of participation in specific activities during the previous year. Each item was measured using an 8-point frequency response scale (1=“almost never,” 2=“about once a year,” 3=“about once every 6 months,” 4=“about once every 3 months,” 5=“about 1-3 times a month,” 6=“about 1-3 times a week,” 7=“about 4-6 times a week,” and 8=“almost daily”). Because these response categories reflect ordered participation frequencies rather than exact event counts, we converted them into estimated annual participation counts to facilitate comparison across activities and domains.

The activity variables were grouped into 3 domains: physical activity (the item labeled “Sports/outdoor activity frequency”), social activity (the items labeled “Friendship/social activity frequency,” “Social/political activity frequency,” and “Religious activity frequency”), and cultural activity (the items labeled “Creative hobby activity frequency” and “Performance/cultural attendance frequency”). For each domain, estimated annual counts were summed across the relevant items. An overall health-related lifestyle activity measure was calculated by summing counts across all domains. These 6 survey items were included in the 2016 survey as questions 17‐1 through 17‐6 and in the 2022 survey as questions 29‐1 through 29‐6 but were not included in the 2017 survey. Accordingly, the 2016 lifestyle activity measures represented the most recent available outcome measurements preceding the 2017 wearable ownership assessment.

### Covariates

To address potential confounding, we adjusted for demographic and socioeconomic characteristics that may be associated with both wearable device adoption and health-related lifestyle behaviors. These covariates included age, sex, educational attainment, household income, household composition, and region of residence. Age was categorized as 20 to 39 years, 40 to 64 years, and 65 years and above. Educational attainment was categorized as middle school or lower vs high school or higher. Household income was grouped into 3 categories: low (<KRW 2 million [US $1357.24] per month), middle (KRW 2-3.99 million [US $1357.24-$2707.69] per month), and high (≥KRW 4 million [US $2714.48] per month). The “metropolitan” region of residence referred to the capital region of South Korea (Seoul and its surrounding metropolitan areas) as well as major metropolitan cities, whereas “nonmetropolitan” included all other cities and provinces outside these metropolitan regions. These variables were selected a priori based on prior literature and conceptual considerations related to social participation, access to digital technologies, and health-related behavior [14].

### Statistical Analysis

Baseline sociodemographic characteristics were summarized according to wearable device adoption status. Frequencies and percentages were reported, and standardized differences were calculated to assess baseline imbalance between adopters and nonadopters. We used a difference-in-differences (DID) framework [15] to examine whether changes in health-related lifestyle behaviors from 2016 to 2022 differed between adopters and nonadopters. The DID parameter of interest was the interaction between time (2016 vs 2022) and adoption status (adopter vs nonadopter). Because only 2 waves were available, the parallel trends assumption could not be directly evaluated using preexposure trends.

We fitted generalized estimating equation models to account for repeated observations within individuals. Relative DID estimates were derived from Poisson models using a log link, and adjusted absolute DID estimates for annual activity frequencies were derived from identity link models. Each model included indicators for time, adoption status, and their interaction, with adjustment for age, sex, educational attainment, household income, household composition, and region of residence. Subgroup analyses were conducted by sex, educational attainment, household income, household composition, and region of residence to explore heterogeneity in associations. As a sensitivity analysis, we additionally applied inverse probability of treatment weighting based on baseline covariates to further address observed baseline imbalance between adopters and nonadopters. All analyses were conducted using SAS Enterprise Guide (version 7.1; SAS Institute).

### Ethical Considerations

This nationwide population-based retrospective cohort study was approved by the institutional review board of Severance Hospital, South Korea (4-2025-1151). This study was conducted in accordance with the tenets of the Declaration of Helsinki. Written informed consent was waived because the study used deidentified database data.

## Results

A total of 5507 participants were included in the analysis, of whom 730 (13.3%) were wearable adopters and 4777 (86.7%) were nonadopters. As shown in Table 1, substantial baseline imbalance was observed between adopters and nonadopters, particularly in age, educational attainment, and household income. Adopters were generally younger, more highly educated, and more likely to have a higher household income and live in metropolitan areas.

**Table 1.** General characteristics of study participants (N=5507).

Variable	Wearable device adoption, n (%)^a^			Standardized difference
	Total	Yes (n=730)	No (n=4777)	
Age (y)				1.034
20-39	727 (13.2)	265 (36.3)	462 (9.7)	
40-64	2945 (53.5)	443 (60.7)	2502 (52.4)	
≥65	1835 (33.3)	22 (3.0)	1813 (38.0)	
Sex				0.134
Male	2360 (42.9)	355 (48.6)	2005 (42.0)	
Female	3147 (57.1)	375 (51.4)	2772 (58.0)	
Educational attainment				0.885
Middle school or lower	1524 (27.7)	10 (1.4)	1514 (31.7)	
High school or higher	3983 (72.3)	720 (98.6)	3263 (68.3)	
Household income^b^				0.638
Low	1425 (25.9)	51 (7.0)	1374 (28.8)	
Middle	2253 (40.9)	316 (43.3)	1937 (40.5)	
High	1829 (33.2)	363 (49.7)	1466 (30.7)	
Region of residence^c^				0.352
Metropolitan	3312 (60.1)	543 (74.4)	2769 (58.0)	
Nonmetropolitan	2195 (39.9)	187 (25.6)	2008 (42.0)	
Household composition				0.173
One-person household	502 (9.1)	26 (3.6)	476 (10.0)	
Multiperson household	5005 (90.9)	704 (96.4)	4301 (90.0)	

a Participants who already owned a wearable device in 2017 were excluded from the analytic sample. “Yes” refers to individuals who did not own a wearable device in 2017 but owned one in 2022, whereas “No” refers to those who did not own a wearable device in either 2017 or 2022.

b Household income was grouped into 3 categories: low (<KRW 2 million [US $1357.24] per month), middle (KRW 2‐3.99 million [US $1357.24-$2707.69] per month), and high (≥KRW 4 million [US $2714.48] per month).

c “Metropolitan” refers to the capital region of South Korea (Seoul and its surrounding metropolitan areas) as well as major metropolitan cities. “Nonmetropolitan” includes all other cities and provinces outside these metropolitan regions.

Table 2 shows both absolute DID estimates expressed as annual frequency differences and relative DID estimates expressed as rate ratios (RRs). In the overall analysis, wearable adoption was associated with greater increases in total health-related lifestyle behavior over time (absolute DID=29.13, 95% CI 15.42-42.84; RR 1.24, 95% CI 1.08-1.35). For domain-specific outcomes, wearable adoption was associated with increases in physical activity and cultural activity but not social activity. The adjusted absolute DID estimate for physical activity was 22.88 (95% CI 14.92-30.84), corresponding to an RR of 1.36 (95% CI 1.12-1.64). For cultural activity, the adjusted absolute DID estimate was 5.45 (95% CI 0.55-10.36), with an RR of 1.78 (95% CI 1.31-2.42). No clear association was observed for social activity (absolute DID=0.74, 95% CI −7.27 to 8.75; RR 1.03, 95% CI 0.91-1.16). In sensitivity analyses using inverse probability of treatment weighting (IPTW), the overall pattern of results was broadly similar to that of the primary analyses (Multimedia Appendix 1).

**Table 2.** Changes in health-related lifestyle behaviors after wearable device adoption.

Outcome	Nonadopters^a^, mean annual frequency		Adopters, mean annual frequency		Absolute change^b^ (frequency)		Relative change (rate ratio)	
	2016 wave	2022 wave	2016 wave	2022 wave	Adjusted difference-in-differences estimate (95% CI)	*P* value	Adjusted difference-in-differences estimate (95% CI)	*P* value
Total health-related lifestyle behavior^c^								
Overall	85.24	91.69	113.42	152.62	29.13 (15.42 to 42.84)	<.001	1.24 (1.08 to 1.35)	<.001
20-39 y	95.52	116.59	119.16	154.21	17.19 (−7.29 to 41.68)	.17	1.08 (0.89 to 1.31)	.45
40-64 y	86.52	95.00	107.65	152.08	35.24 (16.75 to 53.74)	<.001	1.30 (1.11 to 1.52)	<.001
≥65 y	75.98	80.77	71.01	144.34	67.52 (−52.98 to 188.02)	.27	1.80 (0.49 to 6.60)	.38
Physical activity								
Overall	20.39	32.16	30.78	67.21	22.88 (14.92 to 30.84)	<.001	1.36 (1.12 to 1.64)	.002
20-39 y	22.62	39.53	31.00	65.59	19.44 (5.55 to 33.32)	.006	1.27 (0.91 to 1.78)	.16
40-64 y	23.05	34.69	30.92	67.76	25.02 (14.70 to 35.34)	<.001	1.50 (1.17 to 1.92)	.001
≥65 y	13.59	26.79	8.33	75.68	55.34 (0.94 to 109.74)	.046	4.30 (0.96 to 19.18)	.06
Social activity								
Overall	52.56	52.39	65.15	66.87	0.74 (−7.27 to 8.75)	.86	1.03 (0.91 to 1.16)	.68
20-39 y	50.64	59.76	67.55	69.66	−5.89 (−19.59 to 7.81)	.40	0.88 (0.71 to 1.09)	.24
40-64 y	51.19	52.08	62.55	65.50	1.30 (−10.01 to 12.62)	.82	1.02 (0.85 to 1.23)	.81
≥65 y	56.58	50.95	57.85	60.83	7.96 (−67.37 to 83.29)	.84	1.14 (0.33 to 3.96)	.84
Cultural activity								
Overall	12.28	7.14	17.49	18.54	5.45 (0.55 to 10.36)	.03	1.78 (1.31 to 2.42)	<.001
20-39 y	22.26	17.30	20.60	18.96	3.59 (−4.68 to 11.86)	.40	1.18 (0.76 to 1.84)	.47
40-64 y	12.28	8.23	14.18	18.82	8.83 (1.75 to 15.91)	.02	2.05 (1.28 to 3.27)	.003
≥65 y	5.81	3.03	4.83	7.83	4.74 (−8.63 to 18.12)	.49	2.69 (0.39 to 18.58)	.32

a Because all participants included in the analytic cohort did not own a wearable device in 2017, group assignment was determined based on wearable ownership in 2022. Nonadopters comprised individuals who did not own a wearable device in either 2017 or 2022, whereas adopters included those who transitioned from nonownership in 2017 to ownership in 2022.

b Absolute change indicates the annual difference in differences in the number of activity engagements, whereas relative percentage change reflects the proportional difference-in-differences effect expressed as a percentage. All estimates were adjusted for age, sex, educational attainment, household composition, household income, and region of residence.

c Health-related lifestyle behaviors refer to physical, social, and cultural activity. Physical activity refers to participation in sports, exercise, or outdoor recreational activities. Social activity includes friendship-based gatherings, religious participation, and engagement in community or sociopolitical groups. Cultural activity encompasses hobby-related activities and attendance to cultural events such as performances, exhibitions, or other arts-related experiences.

Table 3 presents subgroup-specific relative DID estimates and formal tests for interaction. Overall, evidence of interaction was limited and inconsistent across domains, although some heterogeneity was observed for region of residence and educational attainment. Given the wide CIs and sparse data in several strata, these subgroup findings should be regarded as exploratory.

**Table 3.** Difference-in-differences estimates of health-related lifestyle behaviors by demographic characteristic.

Outcome	Total health promotion activity^a^ rate ratio^b^ (95% CI)	*P* value for the interaction	Social activity rate ratio (95% CI)	*P* value for the interaction	Cultural activity rate ratio (95% CI)	*P* value for the interaction	Physical activity rate ratio (95% CI)	*P* value for the interaction
Sex		.34		.38		.76		.25
Male	1.17 (0.99‐1.38)		0.96 (0.79‐1.17)		1.88 (1.15‐3.08)		1.23 (0.97‐1.57)	
Female	1.30 (1.12‐1.51)		1.08 (0.92‐1.26)		1.72 (1.17‐2.54)		1.53 (1.14‐2.07)	
Household income^c^		.23		.40		.18		.54
Low	1.85 (1.07‐3.21)		1.48 (0.70‐3.14)		8.48 (2.18‐32.93)		1.64 (0.41‐6.59)	
Middle-high	1.23 (1.09‐1.37)		0.998 (0.88‐1.13)		1.70 (1.24‐2.33)		1.39 (1.14‐1.69)	
Region of residence^d^		<.001		.007		.07		.01
Metropolitan	1.08 (0.95‐1.23)		0.87 (0.75‐1.01)		1.54 (1.09‐2.18)		1.28 (1.03‐1.60)	
Nonmetropolitan	1.78 (1.43‐2.21)		1.39 (1.10‐1.76)		2.77 (1.42‐5.40)		1.95 (1.31‐2.90)	
Educational attainment		.02		.04		.23		.07
Middle school or lower	5.52 (1.54‐19.80)		3.84 (1.19‐12.36)		123.28 (25.84‐588.16)		6.80 (1.21‐38.38)	
High school or higher	1.23 (1.10‐1.38)		0.99 (0.87‐1.12)		1.62 (1.19‐2.22)		1.48 (1.23‐1.80)	
Household composition		.17		.34		.006		.74
Single person	1.90 (1.17‐3.08)		1.43 (0.74‐2.76)		16.77 (6.95‐40.49)		1.86 (0.80‐4.35)	
Nonsingle person	1.22 (1.09‐1.37)		1.01 (0.89‐1.15)		1.64 (1.20‐2.25)		1.36 (1.12‐1.65)	

a Health-related lifestyle behaviors refer to physical, social, and cultural activity. Physical activity refers to participation in sports, exercise, or outdoor recreational activities. Social activity includes friendship-based gatherings, religious participation, and engagement in community or sociopolitical groups. Cultural activity encompasses hobby-related activities and attendance to cultural events such as performances, exhibitions, or other arts-related experiences.

b Rate ratio indicates the adjusted relative difference-in-differences (DID) effect within each subgroup. The *P* value for the interaction tests whether the DID effect differs between subgroup categories. All estimates were adjusted for age, sex, educational attainment, household composition, household income, and region of residence.

c Household income was grouped into 2 categories: low (<KRW 2 million [US $1357.24] per month) and middle-high (≥KRW 2 million [US $1357.24] per month).

d “Metropolitan” refers to the capital region of South Korea (Seoul and its surrounding metropolitan areas) as well as major metropolitan cities. “Nonmetropolitan” includes all other cities and provinces outside these metropolitan regions.

## Discussion

### Principal Findings

In this longitudinal study using a DID framework, wearable device adoption was associated with greater increases in total, physical, and cultural health-related lifestyle behaviors over time, whereas no clear association was observed for social activity. Overall, these findings are broadly consistent with those of prior studies reporting favorable findings regarding wearable devices and physical activity [5,16,17]. However, our study extends this literature by examining wearable adoption in a real-world population setting using observational panel data rather than short-term intervention-based or highly controlled study designs. Several possible explanations may account for the observed associations, although these mechanisms were not directly measured in this study. Prior literature suggests that wearable devices may support self-monitoring, goal setting, and feedback, which in turn may be related to greater engagement in some health-related behaviors [10,18,19]. At the same time, the absence of a clear association with social activity may reflect the fact that many social activities, such as friendship gatherings, religious participation, and volunteering, depend more heavily on interpersonal, contextual, and community-level factors than on individual self-tracking tools [7,20].

In age-stratified analyses, associations were most consistently observed among adults aged 40 to 64 years, whereas estimates among older adults were less precise and should be interpreted cautiously. One possible interpretation is that wearable adoption may be more strongly linked to behavior change among individuals facing greater barriers to physical activity or greater interest in health monitoring in midlife and later life [6,17]. However, several subgroup estimates, particularly among older adults, were based on relatively small numbers of adopters and were accompanied by wide CIs. Accordingly, these findings should be viewed as exploratory rather than definitive. Subgroup analyses otherwise showed limited and inconsistent evidence of heterogeneity across sociodemographic characteristics. Interaction tests suggested some heterogeneity for region of residence and educational attainment in selected outcomes, but most interaction tests were not statistically significant. Given the wide CIs in several strata, these subgroup findings should not be overinterpreted.

Our findings suggest that wearable adoption may be associated with favorable changes in some lifestyle-related activities in a real-world setting. At the same time, wearable adoption in this study was voluntary and strongly socially patterned: adopters were younger, more highly educated, and more likely to have a higher household income and live in metropolitan areas than nonadopters. Accordingly, the observed associations may partly reflect selection processes related to digital literacy, socioeconomic advantage, or other unmeasured factors rather than wearable adoption itself. These issues should be considered carefully when interpreting the findings.

### Limitations

This study has several limitations. First, only 2 survey waves (2016 and 2022) were available, which prevented direct evaluation of the parallel trends assumption underlying the DID framework. This is an important limitation because covariate adjustment cannot verify whether adopters and nonadopters would have followed similar preadoption trajectories in the absence of wearable adoption. Therefore, the findings should be interpreted as associative rather than causal. Second, substantial baseline imbalance was observed between adopters and nonadopters, particularly with respect to age, educational attainment, and household income. Although we adjusted for measured sociodemographic characteristics, residual confounding and selection bias remain possible. In particular, unmeasured factors during and after the COVID-19 pandemic may have influenced both wearable adoption and changes in activity patterns. Third, wearable device adoption was assessed only at each survey wave, and the survey did not capture the exact timing of adoption, duration of device use, or intensity of engagement. Therefore, participants classified as adopters may have varied in the extent of their actual device use, and the findings should not be interpreted as reflecting sustained wearable device engagement. Fourth, the activity outcomes were based on self-reported past-year frequency categories and were converted into approximate annual participation counts, which may have introduced measurement error. In addition, because both the exposure and outcome measures were self-reported by the same respondents, common method bias is possible. Fifth, the analytic sample was restricted to participants with complete data at both time points. Because exclusions due to nonparticipation or missing data were substantial, selection bias related to study retention cannot be ruled out.

### Conclusions

This nationally representative panel study found that wearable device adoption was associated with greater increases in total, physical, and cultural health-related lifestyle activities over time, whereas no clear association was observed for social activity. The findings suggest that wearable adoption may be linked to favorable behavioral patterns in some domains, warranting further research using designs that can better address selection processes, timing of adoption, and actual device use.

## Supplementary material

10.2196/88276 Multimedia Appendix 1 Comparison of primary and inverse probability of treatment–weighted difference-in-differences estimates for health-related lifestyle behaviors.

10.2196/88276 Checklist 1 STROBE checklist.

## Abbreviations

DID: difference-in-differences
RR: rate ratio
STROBE: Strengthening the Reporting of Observational Studies in Epidemiology
