# Correction: Association Between Wearable Device Adoption and Health-Related Lifestyle Behaviors: Retrospective Cohort Study

**DOI:** 10.2196/112656

**Published:** 2026-10-28

**Authors:** Eunjeong Choi, Seoyeong Choi, Suk-Yong Jang

**Affiliations:** 1 Department of Public Health, Graduate School, Yonsei University, Seoul, Republic of Korea; 2 Department of Biohealth Industry, Policy Analysis Division, Graduate School of Transdisciplinary Health Science, Yonsei University, 50 Yonsei-ro, Seodaemun-gu, Seoul, 03722, Republic of Korea, 82 2 2228 1511, 82 2 392 7734; 3 Department of Health Policy and Management, Graduate School of Public Health, Yonsei University, Seoul, Republic of Korea

In “Association Between Wearable Device Adoption and Health-Related Lifestyle Behaviors: Retrospective Cohort Study” [1], the temporal alignment between the lifestyle outcome measurements and wearable device ownership assessments was described incorrectly in several sections. Lifestyle activity outcomes were measured in 2016 and 2022, whereas wearable device ownership was assessed in 2017 and 2022. Wearable nonownership used for exposure classification was ascertained in 2017. Accordingly, the following corrections have been made.

In the Methods section of the Abstract the following sentence was corrected:

*We analyzed data from the 2016 and 2022 waves of the Korea Media Panel survey.*

The sentence now reads:

*We analyzed lifestyle activity outcomes from the 2016 and 2022 waves and wearable device ownership from the 2017 and 2022 waves of the Korea Media Panel survey.*

In the Data Source section of the Methods, the following sentence was replaced:

*This study used data from the 2016 and 2022 waves of the Korea Media Panel survey.*

The sentence now reads:

*This study used data from the 2016, 2017, and 2022 waves of the Korea Media Panel survey. Lifestyle activity outcomes were measured in 2016 and 2022, whereas wearable device ownership was assessed in 2017 and 2022.*

In the Study Population section of the Methods, the following sentence was revised:

*We used the 2016 wave as baseline and the 2022 wave as follow-up.*

The sentence now reads:

*We used the 2016 and 2022 waves as the baseline and follow-up measurements, respectively, for the lifestyle activity outcomes, whereas wearable device ownership in 2017 was used to establish wearable nonownership before potential adoption.*

In the same paragraph, the following phrase was revised:

*those who reported wearable device use at baseline (n=132, 1.5%)*

The phrase now reads:

*those who reported wearable device ownership in 2017 (n=132, 1.5%).*

In the Exposure Variable section of the Methods, the following paragraph was revised:

*The exposure of interest was new wearable device adoption between 2016 and 2022. Participants were classified as adopters if they did not report wearable device use in 2016 but reported wearable device use in 2022. Participants who did not report wearable device use in either wave were classified as nonadopters.*

The paragraph now reads:

*The exposure of interest was new wearable device adoption between 2017 and 2022. Participants were classified as new adopters if they did not own a wearable device in 2017 but reported wearable device ownership in 2022. Participants who did not own a wearable device in either 2017 or 2022 were classified as nonadopters.*

In the Outcome Variables section of the Methods, the following paragraph was revised:

*The activity variables were grouped into 3 domains: physical activity (sports or outdoor activities), social activity (friendship interactions, social or political participation, and religious activities), and cultural activity (creative hobbies and attendance to cultural events). For each domain, estimated annual counts were summed across the relevant items. An overall health-related lifestyle activity measure was calculated by summing counts across all domains.*

The paragraph now reads:

*The activity variables were grouped into 3 domains: physical activity (the item labeled “Sports/outdoor activity frequency”), social activity (the items labeled “Friendship/social activity frequency,” “Social/political activity frequency,” and “Religious activity frequency”), and cultural activity (the items labeled “Creative hobby activity frequency” and “Performance/cultural attendance frequency”). For each domain, estimated annual counts were summed across the relevant items. An overall health-related lifestyle activity measure was calculated by summing counts across all domains. These 6 survey items were included in the 2016 survey as questions 17‐1 through 17‐6 and in the 2022 survey as questions 29‐1 through 29‐6 but were not included in the 2017 survey. Accordingly, the 2016 lifestyle activity measures represented the most recent available outcome measurements preceding the 2017 wearable ownership assessment.*

Footnote a in Table 1 was revised from the following:

*Participants who already owned a wearable device at baseline (2016) were excluded from the analytic sample. “Yes” refers to individuals who did not own a wearable device at baseline but owned one by the follow-up in 2022, whereas “No” refers to those who never owned a device during the study period.*

The footnote now reads:

*Participants who already owned a wearable device in 2017 were excluded from the analytic sample. “Yes” refers to individuals who did not own a wearable device in 2017 but owned one in 2022, whereas “No” refers to those who did not own a wearable device in either 2017 or 2022.*

Footnote a in Table 2 was revised from the following:

*Because all participants were nonadopters at baseline (2016), group assignment was determined based on wearable adoption at follow-up (2022). Nonadopters comprised individuals who remained nonadopters in both 2016 and 2022, whereas adopters included those who newly adopted a wearable device by 2022.*

The footnote now reads:

*Because all participants included in the analytic cohort did not own a wearable device in 2017, group assignment was determined based on wearable ownership in 2022. Nonadopters comprised individuals who did not own a wearable device in either 2017 or 2022, whereas adopters included those who transitioned from nonownership in 2017 to ownership in 2022.*

Statements referring to only 2 available survey waves have been clarified to indicate that only 2 waves of lifestyle activity outcome measurements, in 2016 and 2022, were available for the difference-in-differences analysis.

These corrections clarify the temporal alignment between the lifestyle outcome measurements and wearable device ownership assessments, the definition of new wearable adopters, and the specific survey items used to construct the lifestyle activity outcome domains. These corrections do not affect the analyses, reported results, or conclusions of the study.

The correction will appear in the online version of the paper on the JMIR Publications website together with the publication of this correction notice. Because this was made after submission to PubMed, PubMed Central, and other full-text repositories, the corrected article has also been resubmitted to those repositories.
