# Supplementary material for: Association Between Wearable Device Adoption and Health-Related Lifestyle Behaviors: Retrospective Cohort Study
Source: J Med Internet Res. 2026 May 25;28:e88276. doi: 10.2196/88276 (PMC13200801; doi:10.2196/88276)
Supplement: Multimedia Appendix 1 [file jmir-v28-e88276-s001.docx]

**Supplementary Table 1. Comparison of primary and IPTW-weighted difference-in-differences estimates for health-related lifestyle behaviors**

|  | **Relative change (rate ratio)** | | | |
| --- | --- | --- | --- | --- |
|  | **Adjusted difference- in-differenced (95% CI)** | **P value** | **IPTW-weighted Adjusted difference- in-differenced (95% CI)** | **P value** |
| **Outcome** |  |  |  |  |
| **Total health-related lifestyle behavior** |  |  |  |  |
| Overall | 1.24 (1.08 – 1.35) | <.001 | 1.29 (1.14-1.45) | <.001 |
| 20 to 39 | 1.08 (0.89 – 1.31) | 0.453 | 1.10 (0.90-1.34) | 0.365 |
| 40 to 64 | 1.30 (1.11 – 1.52) | <.001 | 1.32 (1.12-1.54) | <.001 |
| 65 and over | 1.80 (0.49 – 6.60) | 0.378 | 1.88 (0.53-6.69) | 0.330 |
| **Physical activity** |  |  |  |  |
| Overall | 1.36 (1.12 – 1.64) | 0.002 | 1.50 (1.23-1.83) | <.001 |
| 20 to 39 | 1.27 (0.91 – 1.78) | 0.159 | 1.31 (0.93-1.85) | 0.121 |
| 40 to 64 | 1.50 (1.17 -1.92) | 0.001 | 1.61 (1.26-2.07) | <.001 |
| 65 and over | 4.30 (0.96 – 19.18) | 0.056 | 4.46 (1.08-18.40) | 0.039 |
| **Social activity** |  |  |  |  |
| Overall | 1.03(0.91 – 1.16) | 0.68 | 1.03 (0.90-1.18) | 0.698 |
| 20 to 39 | 0.88 (0.71 – 1.09) | 0.236 | 0.90 (0.73-1.12) | 0.341 |
| 40 to 64 | 1.02 (0.85 – 1.23) | 0.811 | 1.01 (0.84-1.21) | 0.921 |
| 65 and over | 1.14 (0.33 – 3.96) | 0.841 | 1.17 (0.34-4.02) | 0.809 |
| **Cultural activity** |  |  |  |  |
| Overall | 1.78 (1.31 – 2.42) | <.001 | 1.77 (1.25-2.51) | 0.001 |
| 20 to 39 | 1.18 (0.76 – 1.84) | 0.471 | 1.16 (0.74-1.82) | 0.509 |
| 40 to 64 | 2.05 (1.28 – 3.27) | 0.003 | 1.95 (1.20-3.17) | 0.007 |
| 65 and over | 2.69 (0.39 – 18.58) | 0.316 | 3.26 (0.46-22.93) | 0.236 |

Relative change estimates are presented as adjusted difference-in-differences rate ratios (RRs) with 95% confidence intervals. IPTW indicates inverse probability of treatment weighting.
